# An Epidemiological Meta-Analysis on the Worldwide Prevalence, Resistance, and Outcomes of Spontaneous Bacterial Peritonitis in Cirrhosis

**DOI:** 10.3389/fmed.2021.693652

**Published:** 2021-08-05

**Authors:** Phoebe Wen Lin Tay, Jieling Xiao, Darren Jun Hao Tan, Cheng Ng, Yan Nerng Lye, Wen Hui Lim, Vanessa Xin Yi Teo, Ryan Rui Yang Heng, Marcus Wei Xuan Yeow, Lionel Hon Wai Lum, Eunice Xiang Xuan Tan, Guan Sen Kew, Guan Huei Lee, Mark D. Muthiah

**Affiliations:** ^1^Yong Loo Lin School of Medicine, National University Singapore, Singapore, Singapore; ^2^Division of Infectious Diseases, Department of Medicine, National University Hospital, Singapore, Singapore; ^3^Division of Gastroenterology and Hepatology, Department of Medicine, National University Hospital, Singapore, Singapore; ^4^National University Centre for Organ Transplantation, National University Hospital, Singapore, Singapore

**Keywords:** chronic liver disease, cirrhosis, SBP, infection, socioeconomic status

## Abstract

**Background and Aims:** Spontaneous bacterial peritonitis (SBP) is a common and potentially fatal complication of liver cirrhosis. This study aims to analyze the prevalence of SBP among liver cirrhotic patients according to geographical location and income level, and risk factors and outcomes of SBP.

**Methods:** A systematic search for articles describing prevalence, risk factors and outcomes of SBP was conducted. A single-arm meta-analysis was performed using generalized linear mix model (GLMM) with Clopper-Pearson intervals.

**Results:** Ninety-Nine articles, comprising a total of 5,861,142 individuals with cirrhosis were included. Pooled prevalence of SBP was found to be 17.12% globally (CI: 13.63–21.30%), highest in Africa (68.20%; CI: 12.17–97.08%), and lowest in North America (10.81%; CI: 5.32–20.73%). Prevalence of community-acquired SBP was 6.05% (CI: 4.32–8.40%), and 11.11% (CI: 5.84–20.11%,) for healthcare-associated SBP. Antibiotic-resistant microorganisms were found in 11.77% (CI: 7.63–17.73%) of SBP patients. Of which, methicillin-resistant Staphylococcus aureus was most common (6.23%; CI: 3.83–9.97%), followed by extended-spectrum beta-lactamase producing organisms (6.19%; CI: 3.32–11.26%), and lastly vancomycin-resistant enterococci (1.91%; CI: 0.41–8.46%). Subgroup analysis comparing prevalence, antibiotic resistance, and outcomes between income groups was conducted to explore a link between socioeconomic status and SBP, which revealed decreased risk of SBP and negative outcomes in high-income countries.

**Conclusion:** SBP remains a frequent complication of liver cirrhosis worldwide. The drawn link between income level and SBP in liver cirrhosis may enable further insight on actions necessary to tackle the disease on a global scale.

## Introduction

Spontaneous bacterial peritonitis (SBP) is the development of bacterial infection in the peritoneum, with no apparent intra-abdominal source of infection (1). According to the American Association for the Study of Liver Diseases (AASLD) practice guidelines (2), diagnosis of SBP is made in the presence of elevated ascitic fluid polymorphonuclear (PMN) leukocyte count ≥250 cells per mm^3^, although infection can also be diagnosed when patients are ascitic fluid culture positive with PMN <250 cells per mm^3^. SBP is the most common bacterial infection of cirrhosis and ascites with an estimated prevalence of 10–30% (3), and accounts for 4% of cirrhosis-related emergency department visits (4–8). However, outcomes of SBP in cirrhotic patients remain unfavorable. Patients with SBP have elevated risks of developing hepatic encephalopathy, sepsis and renal failure resulting in mortality rates twice that of their uninfected cirrhotic counterparts (9–11).

The exact pathophysiological mechanisms underlying SBP in cirrhosis remain uncertain. It has been theorized to be the result of intestinal bacterial translocation, evidenced by the frequent isolation of gram-negative enteric organisms in patients' ascitic fluid (7, 12). Impaired host immunity and gut microbiome changes such as overgrowth and dysbiosis have been identified as major contributory factors (4, 13). This abnormal gram-negative bacterial overgrowth is suspected to be the consequence of diminished intraluminal bile salts and small intestine mobility (3). Structural abnormalities in the intestinal mucosal wall including wider intracellular spaces, edema, and vascular congestion due to portal hypertension may also account for increased intestinal permeability (14). Immune dysfunction involves hypoalbuminemia as a result of portosystemic shunts in cirrhosis, which may result in compromised humoral and cell-specific immunity (11, 15). Severe acute or chronic liver disease is often accompanied by impaired neutrophilic and reticuloendothelial systems, further increasing susceptibility to infection (16).

However, there has yet to be an integrated analysis of prevalence, risk factors and outcomes of SBP in cirrhotic patients across different populations. Thus, this meta-analysis aims to investigate the prevalence of SBP among cirrhotic patients and explore common risk factors and outcomes correlated with SBP. Lastly, we seek to compare the prevalence, antibiotic resistance profile, and outcomes between different income groups.

## Methods

### Search Strategy

This review adheres to Meta-analysis of Observational Studies in Epidemiology (MOOSE) guidelines for its synthesis (17). Articles relating to SBP were searched for via two electronic databases, Medline and Embase, on 13 November 2020. The search strategy utilized was: [(spontaneous adj3 bacterial adj3 peritonitis).tw.] AND [exp Liver Cirrhosis/or ((hepatic or liver) and (fibrosis or cirrhosis or cirrhotic)).tw.] AND [exp incidence/or exp prevalence/or exp epidemiology/or (inciden^*^ or preval^*^ or epidemiol^*^).tw.] All references were imported into EndNote X9 for removal of duplicates, and subsequent screening of titles and abstracts.

### Eligibility and Data Extraction

The main inclusion criteria of articles were the description of the prevalence of SBP in liver cirrhosis. Only articles with a clinical diagnosis of SBP as positive ascitic fluid culture or ascites fluid analysis with PMN ≥250 per mm^3^ were included (10). Articles that were published before the year 2000 or without clear definitions of SBP were excluded. We included studies with designs including retrospective cohorts, prospective cohorts and cross-sectional studies. Only original studies were included. Commentaries, editorials, reviews, and non-English language publications were excluded. Two authors (PT and JX) independently performed the title and abstract sieve and full-text review based on the inclusion criteria. Discrepancies were resolved by consensus, or through the decision of a third independent author.

Relevant data were extracted into a structured proforma, including information such as country of study, continent, income level, patient characteristics, prevalence of SBP, outcomes, ascitic fluid culture, and source of infection. Extraction of information was done by six authors (PT, JX, CN, DT, YL) in blinded pairs. Outcomes of interest included the pooled prevalence of SBP in cirrhotic patients, prevalence of culture-positive SBP, origin of infection (healthcare-associated vs. community-acquired), presence of antibiotic-resistant organisms in culture-positive SBP, risk factors and outcomes associated with SBP. Included studies defined community-acquired SBP as occurring within 72 h of admission to the hospital, and healthcare-associated SBP as occurring in patients who were hospitalized in the preceding 90 days of current admission (18, 19). In studies that reported the presence of antibiotic-resistant organisms, they were classified as having methicillin-resistant Staphylococcus aureus (MRSA), extended-spectrum beta-lactamase (ESBL), or vancomycin-resistant Enterococcus (VRE) where available. The main clinical outcomes of SBP studied in this meta-analysis were overall mortality, in-hospital mortality, 30-day mortality, 90-day mortality, SBP recurrence, and acute kidney injury (AKI). SBP diagnosis included: (i) culture-positive neutrocytic ascites (CPNA), defined as positive ascitic bacteria culture and ascitic fluid PMN ≥250 cells per mm^3^, (ii) culture-negative neutrocytic ascites (CNNA), defined as a negative ascitic culture with PMN ≥250 cells per mm^3^, and (iii) bacterascites (BA), which was defined as a positive ascitic fluid culture with PMN <250 cells per mm^3^ (20).

### Statistical Analysis and Quality Assessment

A single-arm random-effects model was used in all analyses. The generalized linear mix model (GLMM) with Clopper-Pearson intervals to stabilize the variance has been demonstrated to offer the most accurate estimate in single-arm meta-analysis (21). Statistical heterogeneity was assessed via *I*^2^ and Cochran Q test values, where an *I*^2^ value of 25, 50, and 75% represented low, moderate, and high degree of heterogeneity, respectively (22, 23). A Cochran's *Q*-test with *p* ≤ 0.10 was considered significant for heterogeneity. Regardless, all analysis was conducted in random effects model. Publication bias was not assessed in view of the lack of a tool to measure publication bias in single-arm meta-anaylsis (24). To adjust for baseline demographics including age, gender, alcoholic cirrhosis, hepatocellular carcinoma (HCC), non-alcoholic steatohepatitis (NASH), Child-Pugh Score, and Child-Pugh Score Classes A, B, and C on the prevalence of SBP, a univariate fractional logistic model with a robust variance estimator was conducted. A subgroup analysis was also conducted to compare the prevalence of SBP based on individual geographical regions and countries with a subsequent illustration on the global prevalence using the rates from individual countries.

Next, a comparison was made between middle and high-income countries using the World Bank classification (25, 26). The individual proportions of the middle (p1) and high income (p2) countries were pooled for outcomes relating to prevalence of SBP, prevalence of antibiotic-resistant SBP, mortality, in-hospital mortality, 30-day mortality, 90-day mortality, and prevalence of acute kidney injury (AKI). Then risk ratios were calculated from the division of individual groups (p1/p2) with the lower (LCI) and upper (UCI) bound of the confidence interval estimated from the Katz-logarithmic method (27–29). The *p*-value was calculated after a natural log transformation of the relative risk z-score (27). Quality assessment of included studies was conducted with a risk of bias tool by Hoy et al. consisting of 10 items that address both internal and external validity. The studies were categorized into high, moderate, or low risk of bias (30).

$$\begin{array}{l} LCL=RRe^{\left(-1.96\;\times \sqrt{\frac{1-p_{1}}{n_{1}p_{1}}+\frac{1-p_{2}}{n_{2}p_{2}}}\right)}\\ UCL=RRe^{\left(1.96\;\times \sqrt{\frac{1-p_{1}}{n_{1}p_{1}}+\frac{1-p_{2}}{n_{2}p_{2}}}\right)}\\ p\;value=e^{\left(-0.717\;\times \;|\frac{\ln\;RR}{\frac{\ln UCL-\;\ln\;LCL}{2\;\times \;1.96}}|-0.416\;\times \;{\left(\frac{\ln\;RR}{\frac{\ln UCL-\;\ln\;LCL}{2\;\times \;1.96}}\right)}^{2}\right)} \end{array}$$

## Results

### Summary of Included Articles

**Figure 1:**
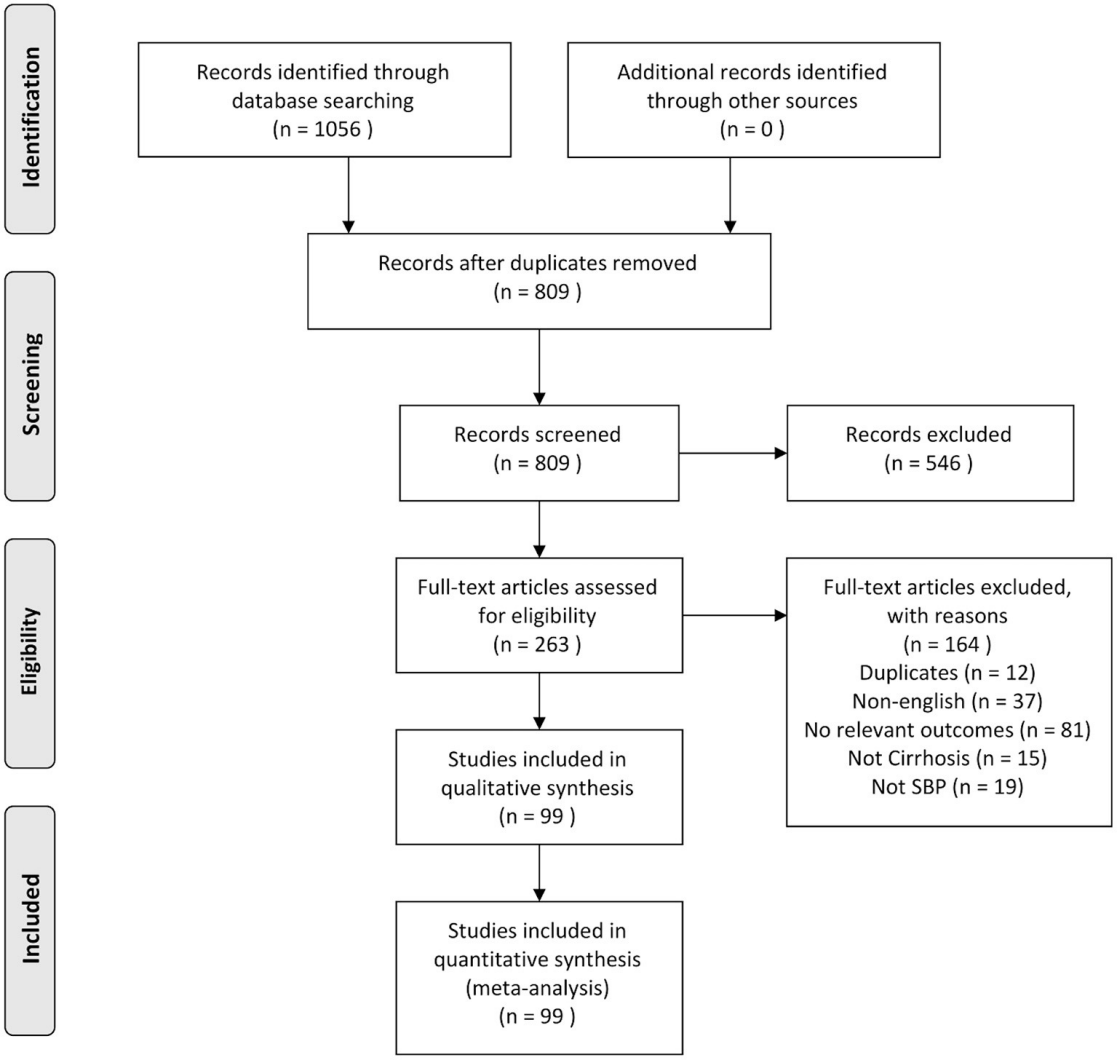
PRISMA flowchart of systematic review.

Using our search strategy, an initial 811 abstracts were identified. Duplicates were removed and 548 articles were excluded based on titles or abstracts. Two hundred sixty-three articles were eligible to undergo full-text review, of which 164 were excluded as they did not meet the inclusion criteria. A detailed description of the selection process is shown in Figure 1. In all, 99 articles were included in the systematic literature review and meta-analysis consisting of a total of 5,861,142 participants. The included studies were conducted across 7 regions, as per World Bank definitions (25), representing 29 countries, with the USA contributing to the largest number of studies (*n* = 11). The studies were from Europe (*n* = 33) (5, 31–61), Asia Pacific (*n* = 21) (62–82), South Asia (*n* = 16) (9, 83–96), North America (*n* = 14) (10, 97–108), Middle East (*n* = 8) (7, 109–115), Latin America (*n* = 5) (116–120), and Africa (*n* = 2) (4, 6). All included papers defined the diagnostic criteria of SBP to be positive ascitic fluid culture or ascitic fluid analysis with PMN count ≥250 cells per mm^3^. A summary of included articles is provided in Supplementary Table 1. Quality assessment shows that majority of the included articles1 had a low (74.8%) or medium (25.3%) risk of bias.

### Prevalence of SBP

**Figure 2:**
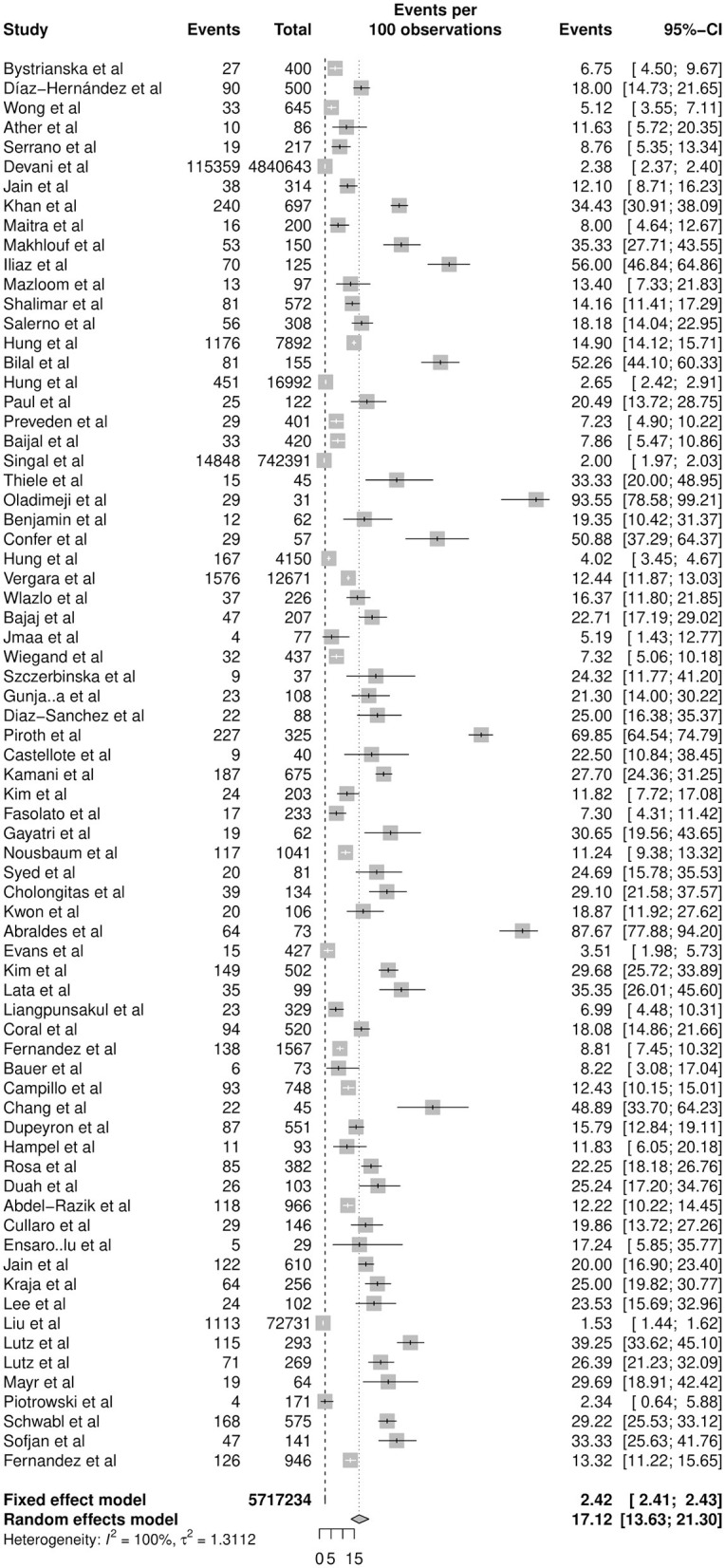
Overall prevalence of SBP in cirrhosis.

In a total of 5,717,234 individuals with cirrhosis, the overall pooled prevalence of SBP was 17.12% (CI: 13.63–21.30%, Figure 2). A fractional regression analysis was then conducted to adjust for risk factors such as age, male gender, alcoholic cirrhosis, hepatocellular carcinoma, Child-Pugh Score, and non-alcoholic steatohepatitis on the prevalence of SBP and summarized in Table 1. The pooled prevalence of community-acquired SBP among patients with cirrhosis was 6.05% (CI: 4.32–8.40%), compared to 11.11% (CI: 5.84–20.11%) for healthcare-associated SBP.

**Table 1:** Risk factors of SBP in cirrhosis.

	**Odds ratio**	**Confidence interval**	***p*-value**
Age	0.9811	0.9435–1.0200	0.336
Gender (male)	0.6337	0.1158–3.4673	0.599
Alcohol	0.9896	0.9821–0.9971	0.473
Hepatitis B	1.2196	0.4102–3.6263	0.721
Hepatitis C	12.4021	2.9523–52.1000	0.001
HCC	0.1062	0.0005–22.0835	0.410
Child–pugh score	0.9836	0.6638–1.4576	0.934
Class A	0.1465	0.0335–0.6412	0.011
Class B	0.1127	0.0001–93.7731	0.525
Class C	5.8597	0.2940–116.7977	0.247
NASH	3.8656	0.1083–137.9366	0.458

**Figure 3:**
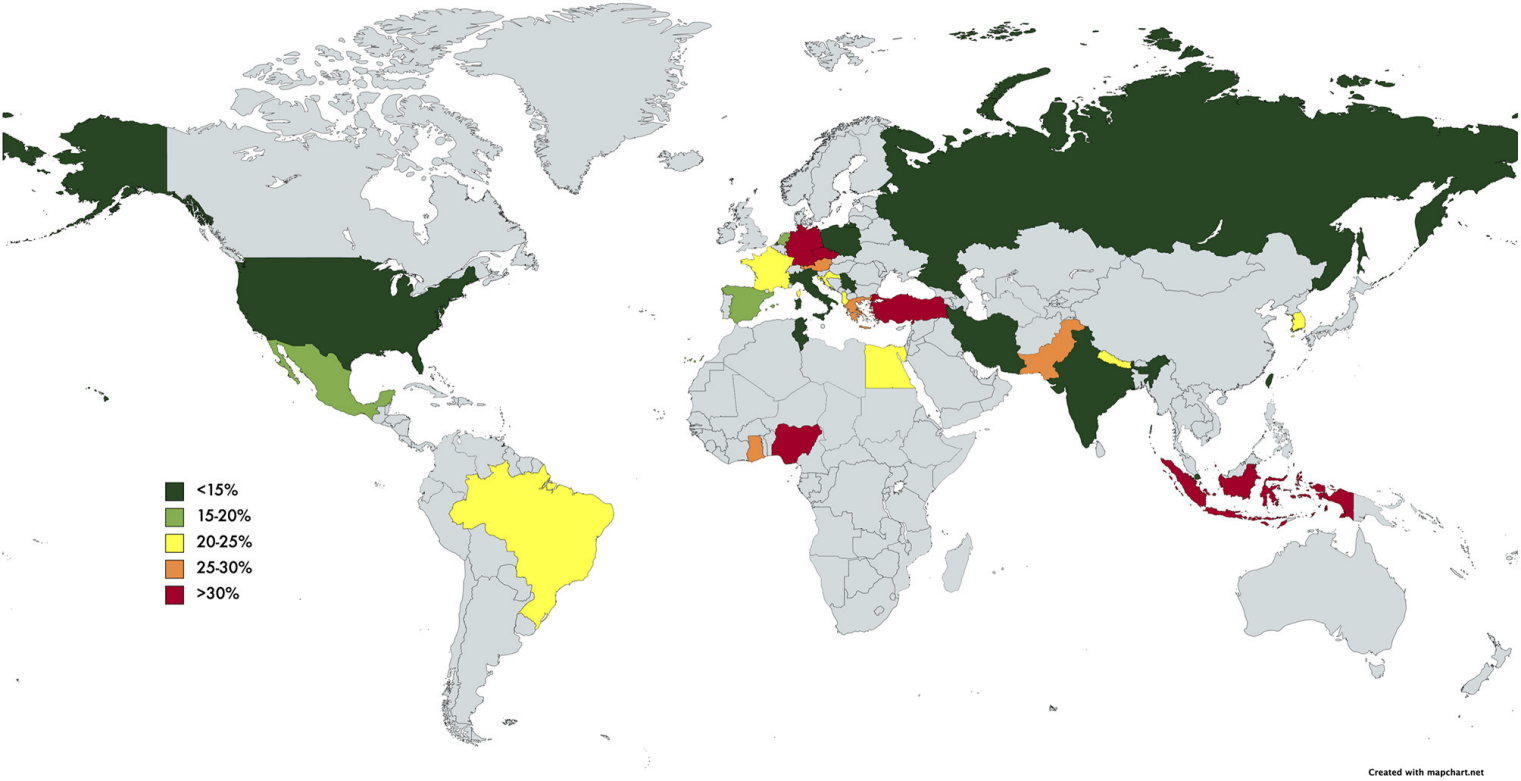
World map of prevalence of SBP in cirrhosis.

The pooled prevalence of SBP of the individual countries was represented in Figure 3. The highest rate of SBP was observed to be in Nigeria (93.55%; CI: 77.58–98.38%) and lowest in Singapore (5.12%; CI: 3.66–7.11%). Subsequently, a subgroup analysis was performed to investigate the prevalence of SBP stratified by geographical locations (Table 2). According to region, Africa had the highest pooled prevalence of 68.20% (CI: 12.17–97.08%), while North America had the lowest (10.81%; CI: 5.32–20.27%). The pooled prevalence of SBP in the Asia Pacific region was 14.08% (CI: 7.70–24.36%), 17.73% (CI: 12.34–24.79%) for South Asia, 19.42% (CI: 9.51–35.59%) for Middle East, 18.40% (CI: 13.12–25.18%) for Europe, and 21.17% (CI: 16.84–26.26%) for Latin America.

**Table 2:** Prevalence by continent, source, and outcomes of SBP in cirrhosis.

	**No. of papers**	**Sample (cirrhosis)**	**Events (SBP)**	**Pooled prevalence (95% CI)**
Overall	99	5,861,142	143,908	–
Overall prevalence	72	5,717,234	138,302	17.12% (13.63–21.30%)
**Continent**				
Africa	2	134	60	44.54% (18.15−0.7442%)
North America	13	5,658,362	131,851	10.81% (5.32–20.73%)
Asia Pacific	16	30,699	2,085	14.08% (7.70–24.36%)
South Asia	10	3,297	625	17.73% (12.34–24.79%)
Middle East	6	1,444	263	19.42% (9.51–35.59%)
Europe	19	22,351	3,229	18.4% (13.12–25.18%)
Latin America	3	947	194	21.17% (16.84–26.26%)
**Source of infection**				
Healthcare associated	4	1,301	132	11.11% (5.84–20.11%)
Community acquired	10	4,611	305	6.05% (4.32–8.40%)
**Outcomes**				
Mortality	21	4,753	1,455	30.61% (23.30–39.06%)
In hospital mortality	14	17,037	3,983	23.38% (15.18–34.22%)
30-day mortality	12	3,719	953	25.64% (22.46–29.11%)
90-day mortality	8	2,515	946	37.64% (31.62–44.08%)
Recurrence	3	394	62	15.74% (12.46–19.67%)
Acute kidney injury	5	116,207	57,383	49.38% (30.76–68.18%)

### Ascitic Culture and Antibiotic Resistance of SBP

From a pooled analysis of 4,608 SBP patients, 39.50% (CI: 31.85–47.71%) of these patients had culture-positive ascitic fluid. In analysis of 3,827 SBP patients, CNNA had a prevalence of 55.85% (CI: 43.25–67.74%). Bacterascites accounted for 23.96% (13.74–38.39%) of SBP diagnosis.

**Figure 4:**
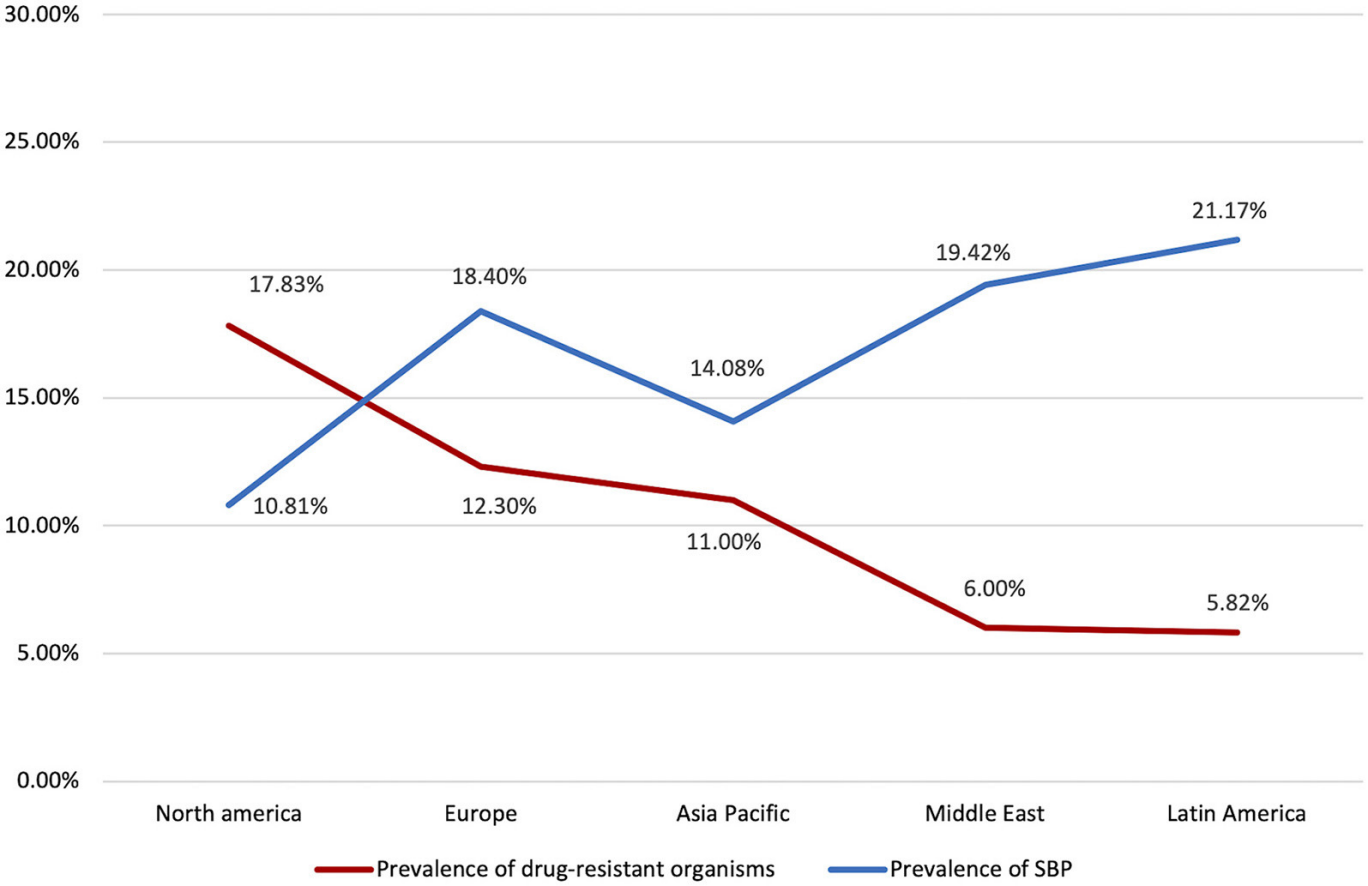
Prevalence of SBP and drug resistance by region.

The distribution of drug-resistant organisms by geographical region is detailed in Table 3 and Figure 4. Antibiotic resistant microorganisms were found in 11.51% (CI: 7.28–17.74%) of SBP patients. In relation to regional differences, North America had the highest prevalence of antibiotic resistant pathogens (17.83%; CI: 2.83–61.73), followed by South Asia (16.78%; CI: 14.23–19.69%), Europe (12.30%; CI: 7.07–20.53%), Asia Pacific (11.0%; CI: 4.65–23.85%), Middle East (6.0%; CI: 3.44–10.27%), and Latin America (5.82%; CI: 3.25–10.20).

**Table 3:** Ascitic culture and antibiotic resistance in SBP.

	**No. of papers**	**Sample (SBP)**	**Events (positive)**	**Pooled prevalence (95% CI)**
**Ascites culture**				
Culture positive	39	4,608	1,844	39.50% (31.85–47.71%)
CNNA	31	3,827	2,149	55.85% (43.25–67.74%)
BA	12	1,542	604	23.96% (13.74–38.39%)
Antibiotic resistant	32	3,263	317	11.77% (7.63–17.73%)
North America	3	329	54	17.83% (2.83–61.73%)
Asia Pacific	3	524	60	11.0% (4.65–23.85%)
Middle East	1	200	12	6.0% (3.44–10.27%)
Latin America	2	189	11	5.82% (3.25–10.20%)
Europe	11	1,300	180	12.3% (7.07–20.53%)
**ESBL**				
Total	12	1,720	129	6.19% (3.32–11.26%)
North America	3	329	35	10.18% (2.34–34.90%)
Asia Pacific	3	524	58	9.88% (4.69–19.65%)
Middle East	1	200	12	6.0% (3.44–10.27%)
Europe	5	667	24	3.35% (1.39–7.84%)
**MRSA**				
Total	10	951	63	6.23%% (3.83–9.97%)
Europe	8	770	54	6.63% (3.70–11.61%)
Latin America	1	134	7	5.22% (2.51–10.55%)
North America	1	47	2	4.26% (1.07–15.48%)
**VRE**				
Total	3	258	4	1.91% (0.41–8.46%)
North America	2	239	2	0.84% (0.21–3.28%)
Europe	1	19	2	10.53% (2.65–33.74%)

*CNNA, culture negative neutrocytic ascites; BA, bacterascites; ESBL, extended spectrum beta-lactamase; MRSA, methicillin-resistant Staphylococcus aureus; VRE, vancomycin-resistant enterococcus*.

Among the resistant organisms reported, MRSA had the highest pooled prevalence (6.23%; CI: 3.83–9.97%), followed by ESBL (6.19%; CI: 3.32–11.26%), and lastly VRE with the lowest reported prevalence (1.91%; CI: 0.41–8.46%). North America had the highest pooled prevalence of ESBL (10.18%; CI: 2.34–34.90%). Europe had the highest pooled prevalence of MRSA and VRE (6.63%; CI: 3.70–11.61%) and (10.53%; CI: 2.65–33.74%), respectively.

### Outcomes in SBP Cirrhosis

**Figure 5:**
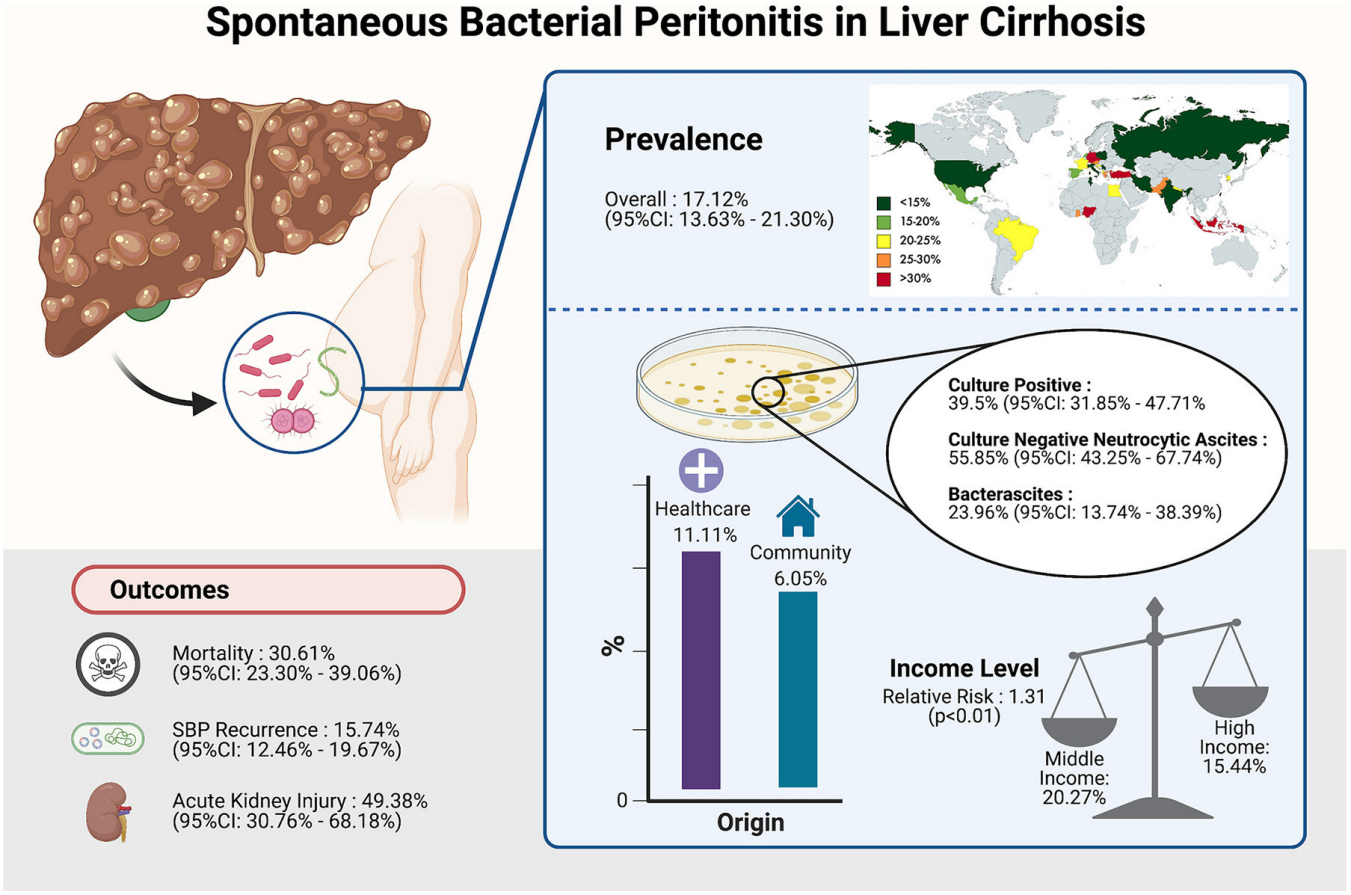
Summary of the prevalence of SBP and its associated outcomes. Image created with Biorender.com.

Incidence of outcomes after SBP infection are summarized in Table 2. The prevalence of SBP and its associated outcomes are illustrated in Figure 5. Overall mortality was 30.61% (CI: 23.30–39.06%), in-hospital mortality (23.38%; CI: 15.18–34.22%), 30-day mortality (25.64%; CI: 22.46–29.11%), 90-day mortality (37.64%; CI: 31.62–44.08%). Acute kidney injury (AKI) had a prevalence of 49.38% (CI: 30.76–68.18%), while SBP recurrence had a prevalence of 15.74% (CI: 12.46–19.67%). Patients with CNNA were observed to have a significantly lower risk of mortality compared to CPNA patients (OR = 0.96; CI: 0.94–0.99; *p* < 0.01). However, no significant difference in mortality was observed between BA and CPNA patients (OR: 1.04, CI: 0.987–1.094).

### Subgroup Analysis on Income Levels

A subgroup analysis of clinical outcomes was conducted according to income level as per World Bank definitions (Table 4). The rate of SBP is significantly higher in patients from middle-income countries (20.27%; CI: 14.89–26.97%) compared to patients from high-income countries (15.44%; CI: 11.29–20.75%) with a relative risk of 1.31 (CI: 1.25–1.37, *p* < 0.01). Middle-income countries were observed to have a higher risk of AKI as a complication (RR = 1.56; CI: 1.47–1.66; *p* < 0.01) than high-income countries. Other outcomes including antibiotic resistance, 30-day mortality and 90-day mortality were observed to be higher in middle-income countries although without significance.

**Table 4:** Prevalence, antibiotic resistance, and outcomes of SBP subgroup by income level.

	**Middle income group**				**Higher income group**						
	**Papers**	**Sample**	**Events**	**Pooled proportion**	**Papers**	**Sample**	**Events**	**Pooled proportion**	**Relative risk**	**95% CI**	***p*-value**
Prevalence	27	7,441	1,366	20.27% (14.89–26.97%)	45	5,709,793	136,936	15.44% (11.29–20.75%)	1.31	1.25–1.37	<0.01
Antibiotic resistance	5	1,334	200	12.65% (7.82–19.81%)	16	1,929	238	11.63% (6.67–19.52%)	1.09	0.90–1.31	0.385
Mortality	11	1,422	434	30.15% (19.39–43.64%)	15	3,331	1,027	30.96% (21.62–42.16%)	0.97	0.89–1.07	0.59
In hospital mortality	4	434	174	22.69% (4.12–66.73%)	10	16,603	3,766	23.47% (16.57–32.15%)	0.97	0.81–1.15	0.72
30-day mortality	4	1,076	269	25.72% (21.65–30.26%)	9	2,645	638	25.58% (21.33–30.36%)	1.01	0.89–1.15	0.94
90-day mortality	1	55	21	38.18% (26.39–51.55%)	7	2,460	949	37.61% (30.95–44.79%)	1.02	0.72–1.43	0.94
AKI	3	355	689	59.68% (33.19–81.52%)	2	115,518	44,117	38.19% (37.91–38.47%)	1.56	1.47–1.66	<0.01

## Discussion

SBP is a common yet debilitating complication of decompensated liver cirrhosis, inflicting a significant global burden (5, 6). This meta-analysis demonstrates the worldwide prevalence, risk factors and outcomes of SBP with notable variation based on different geographical locations and income groups. We found the global pooled prevalence of SBP to be 17.12%, with large variability in prevalence of SBP among geographic regions. The reported prevalence of SBP was four times higher in Africa compared to North America (44.54%; CI 18.15−0.7442 vs. 10.81%; CI 5.32–20.73%, respectively).

Despite aggressive case identification and treatment, SBP remains a significant burden to the healthcare system and was found to have a high mortality rate of 30.61% in our study, comparable to that of variceal bleeding (121). Although this has improved significantly from more than 90% when Harold Conn first described SBP in the 1970s (1), patients with concurrent risk factors are still at a higher risk of mortality. In a large-scale study by Niu et al. which examined 88,167 SBP hospitalizations with 29,963 deaths, it was found that older age, female gender, hepatic encephalopathy, coagulopathy, variceal hemorrhage, sepsis, pneumonia, and acute kidney injury were associated with increased in-patient mortality (122).

Additionally, AKI was found to be a significant outcome of SBP with a prevalence of 49.38%. Renal dysfunction is a common complication of SBP, and a study by Devani et al. demonstrated that there has been an ~2-fold rise of AKI among patients hospitalized with SBP over the past 10 years (98). AKI is also the most important independent predictor of mortality (123) and in a study by Karagozian et al. it was reported that patients with SBP who develop renal failure are more likely to require dialysis and have higher mortality (124).

Interestingly, pooled analysis found that patients with CNNA had significantly lower mortality compared to CPNA patients (OR = 0.96; CI: 0.94–0.99; *p* < 0.01). This is corroborated by previous cohort studies reporting increased mortality in CPNA compared to CNNA patients, attributing this to increased bacterial load in the former (1, 125). Decreased serum and ascitic complement levels, low ascitic fluid protein levels, and reduced activity of the reticulo-endothelial system have been suggested as potential mechanisms for increased severity of ascitic fluid infection in CPNA (126). This could be further confounded by favorable hepatic function in CNNA vs. CPNA patients, as demonstrated by better Child-Pugh scores in the CNNA group reported in previous cohort studies (94, 127). A greater degree of portal hypertension consequent of poorer hepatic function may result in more bacterial translocation, contributing to increased severity of infection, and consequently mortality (94).

This study also reports the overall prevalence of antibiotic resistance in SBP to be 11.51%, with MRSA having the highest prevalence of 6.23%, followed by ESBL (6.19%) and VRE (1.91%). The prevalence of antibiotic-resistant bacteria has been rising due to a global misuse of antibiotics (128), along with the increased frequency of hospitalization and need for invasive procedures in patients with cirrhosis (129). Additionally, rates of resistance varied by continent, which may be a product of varying national antibiotic regimens and policies (130). Proper understanding of resistance rates is essential in determining the appropriate choice of empirical antibiotics, with additional considerations based on the source of infection (61). For community-acquired SBP, the European Association for the Study of the Liver (EASL) recommends that a third-generation cephalosporin be used as the first-line antibiotic treatment in countries with low rates of bacterial resistance, while piperacillin/tazobactam or carbapenem should be reserved for community-acquired cases in countries with high rates of resistance. Due to the increased prevalence of multi-drug resistance in healthcare-associated SBP, the EASL guidelines recommend piperacillin/tazobactam for healthcare-related cases in areas with low prevalence of multi-drug resistance. In regions where multi-drug resistant bacteria are more common, carbapenem in combination with linezolid or glycopeptides can be considered (20).

Interestingly, in regions with lower overall prevalence of SBP such as Europe and North America, there were higher rates of antibiotic resistance (Figure 4). This could possibly be attributed to strong adherence to guideline recommendations for prophylaxis in high-risk patients. Randomized control trials have demonstrated that norfloxacin prophylaxis in patients with increased Child-Pugh scores, impaired renal function, or low ascitic protein count was associated with significantly decreased SBP occurrence and increased short-term survival (10). However, it is possible that inappropriate continuation of prophylaxis in patients with improved clinical condition could give rise to multi-drug resistant organisms. In line with EASL guidelines, adequate risk profiling should be conducted to determine the necessity of SBP prophylaxis in order to ensure proper antibiotic stewardship (20).

Finally, socioeconomic status was found to be a significant factor in SBP development, with significantly higher SBP prevalence in patients from middle-income countries compared to patients from higher-income countries. Patients from middle-income countries also had poorer outcomes in terms of 30-day and 90-day mortality, and occurrence of AKI. However, only the relative risk of AKI reached statistical significance (Table 4). Lower-income levels have previously been associated with more advanced disease due to ill-equipped healthcare facilities and poor health literacy (131). Additionally, poorer nutrition in patients from lower-income countries including vitamin D deficiency (132, 133), and increased time to initiation of antibiotics could have further affected outcomes (131). Antimicrobial resistance was also more prevalent in the middle-income group, although it did not reach statistical significance. A multi-country survey by the World Health Organization (WHO) found antibiotic use to be higher in lower-income countries (134), possibly influencing the higher resistance rates.

### Strengths and Limitations

To our best knowledge, this is the first meta-analysis of global prevalence, associative risk factors and outcomes of SBP in patients with liver cirrhosis. Its strengths include the large sample size and rigor in analysis. It is also the first to report the possible impact of socioeconomic status on SBP. A major limitation of this analysis lies in the heterogeneity of the included studies, based on the *I*^2^ statistic However, larger sample sizes are often associated with increased *I*^2^ in simulation studies (135, 136). Thus, a meta-analysis conducted for prevalence often has a large *I*^2^-value (>90%) due to the larger sample sizes involved (137, 138). Current methods of heterogeneity measures are inaccurate in prevalence based meta-analysis (139). Additionally, there is possible underrepresentation of cohorts from lower-income countries, and regions including Africa and Latin America due to publication bias, hence confounding the actual impact of SBP in such communities. The underrepresentation may also be attributed to the exclusion of non-English studies. However, the absence of professional language translators prevents the inclusion of non-English studies and may cause misinterpretation (140). Current translation software (google scholar) has been found to be ill-equipped for systematic review (141).

### Conclusion

This systematic review is the first to provide an analysis of SBP rates on a global level. It also draws an unprecedented link between socioeconomic status and the prevalence of SBP. The discoveries made in this review may assist in the generation of policies to tackle SBP in liver cirrhosis on a community or even global health level. The investigation of antibiotic-resistant microorganism profile according to region highlights the importance of avoiding antibiotic misuse. Thus, this review may help equip physicians with greater awareness of their local antibiotic-resistant microorganism profile and appropriate treatment.

## Data Availability Statement

The original contributions presented in the study are included in the article/Supplementary Material, further inquiries can be directed to the corresponding author/s.

## Author Contributions

MM, CN, and ET: conceptualization. PT, JX, DT, CN, YL, WL, VT, RH, and MY: data curation. PT, JX, DT, and CN: formal analysis. ET, GK, GL, and MM: supervision. PT, JX, DT, CN, YL, WL, VT, RH, MY, and LL: validation. PT, JX, DT, CN, YL, WL, ET, and MM: writing—original draft. PT, JX, DT, CN, YL, WL, VT, RH, MY, LL, ET, GK, GL, and MM: writing—review and editing. All authors read and gave final approval of the version to be submitted.

## Conflict of Interest

The authors declare that the research was conducted in the absence of any commercial or financial relationships that could be construed as a potential conflict of interest.

## Publisher's Note

All claims expressed in this article are solely those of the authors and do not necessarily represent those of their affiliated organizations, or those of the publisher, the editors and the reviewers. Any product that may be evaluated in this article, or claim that may be made by its manufacturer, is not guaranteed or endorsed by the publisher.

## Abbreviations

AKI: Acute kidney injury
AASLD: American Association for the Study of Liver Diseases
BA: Bacterascites
CPNA: Culture-positive neutrocytic ascites
CNNA: culture-negative neutrocytic ascites
EASL: European Association for the Study of the Liver
ESBL: Extended-spectrum beta-lactamase
HCC: Hepatocellular carcinoma
MOOSE: Meta-analysis of Observational Studies in Epidemiology
MRSA: methicillin-resistant Staphylococcus aureus
NASH: non-alcoholic steatohepatitis
PMN: polymorphonuclear
SBP: Spontaneous bacterial peritonitis
VRE: vancomycin-resistant Enterococcus
WHO: World Health Organisation.
