# Supplementary material for: An Epidemiological Meta-Analysis on the Worldwide Prevalence, Resistance, and Outcomes of Spontaneous Bacterial Peritonitis in Cirrhosis
Source: Front Med (Lausanne). 2021 Aug 5;8:693652. doi: 10.3389/fmed.2021.693652 (PMC8375592; doi:10.3389/fmed.2021.693652)
Supplement: Supplementary file 1 [file Table_1.DOCX]

Supplementary Material

# Supplementary Table 1: Summary of included studies

| **Study** | **Year** | **Country of Origin** | **Continent** | **Income Level** | **Sample Size (Cirrhosis)** | **SBP (Events)** | **Age (years)** | **Gender Male (%)** | **Risk of Bias** |
| --- | --- | --- | --- | --- | --- | --- | --- | --- | --- |
| Bystrianska et al, 2020 | 2020 | Russia | Europe | Upper middle | 400 | 27 | 55.0 | 55.4 | L |
| Díaz-Hernández et al | 2020 | Mexico | North America | Upper middle | 500 | 90 | 56.7 | 48.0 | L |
| Li et al | 2020 | China | Asia Pacific | Upper middle | 418 | 418 | - | - | L |
| Li et al | 2020 | Germany | Europe | High | 184 | 184 | - | - | L |
| Mittal et al | 2020 | India | South Asia | Lower middle | 92 | 92 | - | - | L |
| Sanglodkar et al | 2020 | India | South Asia | Lower middle | 92 | 92 | - | - | L |
| Santoiemma et al | 2020 | USA | North America | High | 314 | 314 | - | - | M |
| Wong et al | 2020 | Singapore | Asia Pacific | High | 645 | 33 | - | - | L |
| Al-Ghamdi et al | 2019 | Saudi Arabia | Middle East | High | 200 | 200 | - | - | M |
| Ardolino et al | 2019 | Israel | Middle East | High | 160 | 160 | - | - | M |
| Ather et al | 2019 | Pakistan | South Asia | Lower middle | 86 | 10 | 50.9 | - | L |
| Serrano et al | 2019 | Spain | Europe | High | 217 | 19 | - | - | M |
| Chen et al | 2019 | Taiwan | Asia Pacific | High | 314 | 314 | - | - | L |
| Devani et al | 2019 | USA | North America | High | 4840643 | 115359 | 59.0 | 61.1 | L |
| Ding et al | 2019 | China | Asia Pacific | Upper middle | 334 | 334 | - | - | L |
| Jain et al | 2019 | India | South Asia | Lower middle | 314 | 38 | 52.9 | 86.3 | L |
| Khan et al | 2019 | USA | North America | High | 697 | 240 | 55.0 | 74.6 | L |
| Maitra et al | 2019 | India | South Asia | Lower middle | 200 | 16 | - | 80.5 | M |
| Makhlouf et al | 2019 | Egypt | Middle East | Lower middle | 150 | 48 | 56.3 | 60.0 | L |
| Sarwar et al | 2019 | Pakistan | South Asia | Lower middle | 31 | 31 | - | - | L |
| Iliaz et al | 2018 | Turkey | Middle East | Upper middle | 125 | 70 | 57.1 | 37.9 | L |
| Marciano et al | 2018 | Argentina | Latin America | Upper middle | 55 | 55 | - | - | L |
| Mazloom et al | 2018 | Iran | Middle East | Upper middle | 110 | 13 | 54.63 | 64.9 | M |
| Melcarne et al | 2018 | Spain | Europe | High | 159 | 159 | - | - | L |
| Ning et al | 2018 | China | Asia Pacific | Upper middle | 600 | 600 | - | - | L |
| Shalimar et al | 2018 | India | South Asia | Lower middle | 572 | 81 | 41.7 | 79.7 | L |
| Salerno et al | 2018 | Italy | Europe | High | 308 | 56 | - | - | L |
| Hung et al | 2016 | Taiwan | Asia Pacific | High | 7892 | 1176 | 59.2 | 69.8 | M |
| Oliveira et al | 2016 | Portugal | Europe | High | 139 | 139 | - | - | M |
| Bilal et al | 2015 | Pakistan | South Asia | Lower middle | 155 | 81 | 38.9 | 77.0 | L |
| Hung et al | 2015 | Taiwan | Asia Pacific | High | 16992 | 451 | 59.6 | 70.8 | L |
| Lim et al | 2015 | UK | Europe | High | 93 | 93 | 57.9 | 69.9 | M |
| Paul et al | 2015 | India | South Asia | Lower middle | 122 | 25 | 50.3 | 85.0 | L |
| Preveden et al | 2015 | Serbia | Europe | Upper middle | 401 | 29 | - | - | M |
| Baijal et al | 2014 | India | South Asia | Lower middle | 420 | 33 | 53.1 | 75.9 | L |
| Chaulk et al | 2014 | canada | North America | High | 192 | 192 | - | - | L |
| Singal et al | 2014 | USA | North America | High | 742391 | 14848 | - | - | L |
| Thiele et al | 2014 | Brazil | Latin America | Upper middle | 45 | 15 | 53.2 | 82.2 | M |
| Oladimeji et al | 2013 | Nigeria | Africa | Lower middle | 31 | 21 | - | - | L |
| Benjamin et al | 2013 | India | South Asia | Lower middle | 62 | 12 | 40.7 | 92.5 | L |
| Bhat et al | 2013 | India | South Asia | Lower middle | 70 | 70 | - | - | L |
| Confer et al | 2013 | USA | North America | Higher | 57 | 29 | 56.3 | 67.0 | M |
| Hung et al | 2013 | Taiwan | Asia Pacific | High | 4150 | 167 | 57.6 | 71.7 | L |
| Vergara et al | 2013 | Spain | Europe | High | 12671 | 1576 | 62.3 | 67.7 | L |
| Wlazlo et al | 2013 | Netherlands | Europe | High | 226 | 37 | 59.2 | 64.7 | M |
| Bajaj et al | 2012 | USA | North America | High | 207 | 47 | 55 | 60.0 | L |
| Jmaa et al | 2012 | Tunisia | Middle East | Lower middle | 77 | 4 | 54 | 70.0 | L |
| Novovic et al | 2012 | Denmark | Europe | High | 187 | 187 | - | - | L |
| Shizuma et al | 2012 | Japan | Asia Pacific | High | 30 | 30 | - | - | M |
| Wiegand et al | 2012 | Germany, France | Europe | High | 437 | 32 | 57.3 | 70.0 | M |
| Szczerbinska et al | 2011 | Poland | Europe | High | 37 | 9 | 56.2 | 83.8 | L |
| Gunjača et al | 2010 | Croatia | Europe | High | 108 | 23 | 57.4 | 68.5 | L |
| Kim et al | 2010 | Korea | Asia Pacific | High | 130 | 130 | - | - | L |
| Diaz-Sanchez et al | 2009 | Spain | Europe | High | 88 | 22 | 55.0 | 80.7 | L |
| Heo et al | 2009 | Korea | Asia Pacific | High | 157 | 157 | - | - | L |
| Piroth et al | 2009 | France | Europe | High | 325 | 227 | - | - | L |
| Castellote et al | 2008 | Spain | Europe | High | 40 | 9 | 63.0 | 57.5 | L |
| Haider et al | 2008 | Pakistan | South Asia | Lower middle | 50 | 50 | - | - | L |
| Kamani et al | 2008 | Pakistan | South Asia | Lower middle | 675 | 187 | - | - | L |
| Kim et al | 2005 | Korea | Asia Pacific | High | 203 | 24 | 52.6 | 68.0 | M |
| Fasolato et al | 2007 | Italy | Europe | High | 233 | 17 | 64.24 | 64.4 | L |
| Gayatri et al | 2007 | Indonesia | Asia Pacific | Upper middle | 62 | 19 | 55.23 | 80.7 | L |
| Nousbaum et al | 2007 | France | Europe | High | 1041 | 117 | 60 | 71.9 | L |
| Syed et al | 2007 | Nepal | Asia Pacific | Lower middle | 81 | 17 | 51.1 | 59.2 | L |
| Cholongitas et al | 2006 | Greece | Europe | High | 134 | 39 | 66.8 | 70.1 | M |
| Wang et al | 2006 | China | Asia Pacific | Upper middle | 195 | 195 | - | - | M |
| Kwon et al | 2005 | Korea | Asia Pacific | High | 106 | 20 | 56.24 | 76.4 | M |
| Abraides et al | 2003 | Spain | Europe | High | 73 | 64 | - | - | L |
| Evans et al | 2003 | USA | North America | High | 427 | 15 | 58.1 | 74.0 | L |
| Kim et al | 2003 | Korea | Asia Pacific | High | 502 | 149 | - | - | L |
| Lata et al | 2003 | Czech Republic | Europe | High | 99 | 35 | 56.9 | 62.6 | L |
| Liangpunsakul et al | 2003 | USA | North America | High | 329 | 23 | 49.0 | 59.0 | L |
| Coral et al | 2003 | Brazil | Latin America | Upper middle | 520 | 94 | 49.0 | 76.6 | L |
| Fernandez et al | 2002 | Spain | Europe | High | 1567 | 138 | 61.0 | 16.0 | L |
| Bauer et al | 2002 | Spain | Europe | High | 73 | 6 | 52.0 | - | L |
| Campillo et al | 2001 | France | Europe | High | 748 | 93 | 54.5 | 64.3 | L |
| Chang et al | 2001 | Taiwan | Asia Pacific | high | 45 | 22 | 57.5 | 67.0 | L |
| Dupeyron et al | 2001 | France | Europe | High | 551 | 87 | 55.0 | 35.9 | L |
| Hampel et al | 2001 | USA | North America | High | 93 | 11 | 56.4 | 98.9 | L |
| Rosa et al | 2000 | Brazil | Latin America | Upper middle | 382 | 85 | 45.8 | 68.1 | M |
| Duah et al | 2019 | Ghana | Africa | Lower middle | 103 | 26 | 43.5 | 56.3 | L |
| Abdel-Razik et al | 2001 | Egypt | Middle east | Lower middle | 966 | 118 | 48.5 | 63.1 | L |
| Cho et al | 2013 | Korea | Asia Pacific | High | 336 | 336 | - | - | L |
| Cullaro et al | 2017 | USA | North America | High | 146 | 29 | 59.2 | 55.4 | M |
| Ensaroğlu et al | 2015 | Turkey | Middle East | Upper middle | 29 | 5 | 46.7 | 72.4 | L |
| Jain et al | 2019 | India | South Asia | Lower middle | 610 | 122 | 50.9 | - | L |
| Kraja et al | 2012 | Albania | Europe | Upper middle | 256 | 64 | 54.5 | 77.7 | L |
| Lee et al | 2016 | Korea | Asia Pacific | High | 102 | 24 | 54.5 | 71.6 | M |
| Liu et al | 2016 | USA | North America | High | 72731 | 1113 | 54.5 | 71.6 | L |
| Luz et al | 2018 | Germany | Europe | High | 293 | 115 | 56.75 | 71.0 | L |
| Lutz et al | 2017 | Germany | Europe | High | 269 | 43 | 60.3 | 65.0 | M |
| Mayr et al | 2020 | Germany | Europe | High | 64 | 19 | 60.0 | 67.2 | L |
| Piotrowski et al | 2019 | Poland | Europe | High | 171 | 4 | - | 56.0 | L |
| Schwabl et al | 2015 | Austria | Europe | High | 575 | 168 | 56.9 | 69.9 | L |
| Sofjan et al | 2018 | USA | North America | High | 141 | 47 | 57.5 | 62.0 | L |
| Tsung et al | 2013 | Korea | Asia Pacific | High | 95 | 82 | 58.5 | - | L |
| Xiong et al | 2018 | China | Asia Pacific | Upper middle | - | 216 | - | 0.7368421053 | L |
| Fernandez et al | 2012 | Spain | Europe | High | 946 | 126 | 60.0 | 63.0- | L |

**Risk of Bias Checklist by Hoy et al**

1. Was the study’s target population a close representation of the national population in relation to relevant variables?

2. Was the sampling frame a true or close representation of the target population?

3. Was some form of random selection used to select the sample, OR was a census undertaken?

4. Was the likelihood of nonresponse bias minimal?

5. Were data collected directly from the subjects (as opposed to a proxy)?

6. Was an acceptable case definition used in the study?

7. Was the study instrument that measured the parameter of interest shown to have validity and reliability?

8. Was the same mode of data collection used for all subjects?

9. Were the numerator(s) and denominator(s) for the parameter of interest appropriate?
